# Estimating the Size and Impact of the Ecological Restoration Economy

**DOI:** 10.1371/journal.pone.0128339

**Published:** 2015-06-17

**Authors:** Todd BenDor, T. William Lester, Avery Livengood, Adam Davis, Logan Yonavjak

**Affiliations:** 1 Department of City and Regional Planning, University of North Carolina, Chapel Hill, NC, United States of America; 2 Ecosystem Investment Partners, Baltimore, MD, United States of America; 3 School of Forestry, Yale University, New Haven, CT, United States of America; Universidad Veracruzana, MEXICO

## Abstract

Domestic public debate continues over the economic impacts of environmental regulations that require environmental restoration. This debate has occurred in the absence of broad-scale empirical research on economic output and employment resulting from environmental restoration, restoration-related conservation, and mitigation actions — the activities that are part of what we term the “restoration economy.” In this article, we provide a high-level accounting of the size and scope of the restoration economy in terms of employment, value added, and overall economic output on a national scale. We conducted a national survey of businesses that participate in restoration work in order to estimate the total sales and number of jobs directly associated with the restoration economy, and to provide a profile of this nascent sector in terms of type of restoration work, industrial classification, workforce needs, and growth potential. We use survey results as inputs into a national input-output model (IMPLAN 3.1) in order to estimate the indirect and induced economic impacts of restoration activities. Based on this analysis we conclude that the domestic ecological restoration sector directly employs ~ 126,000 workers and generates ~ $9.5 billion in economic output (sales) annually. This activity supports an additional 95,000 jobs and $15 billion in economic output through indirect (business-to-business) linkages and increased household spending.

## Introduction

A powerful narrative now permeates efforts to regulate environmental impacts and require restoration of damaged ecosystem functions in the wake of development: restoration is expensive, bad for business, and bad for our economy [1, 2]. For example, the U.S. Chamber of Commerce [3] has cautioned against the “corrosive” economic impacts of environmental regulation and permitting processes, which often include requirements for ecological restoration. The authors of this report argue that permitting processes could endanger jobs and earnings. Polls now demonstrate that the American public widely accepts this idea [4] and arguments against job-killing environmental ‘Green Tape’ are common.

This is not a new phenomenon; early studies on the administrative and compliance costs of environmental protection failed to account for the net benefits of environmental protection, including growth in both private- and public-sector environmental protection jobs [2, 5]. Even criticisms of 2014 efforts to expand the federal government’s Clean Water Act jurisdiction [6] have largely ignored any potential benefits of policy changes [7]. What has been almost entirely missing from these management and regulatory decisions is a detailed accounting of the economic output and jobs that are actually created through environmental restoration, restoration-related conservation, and mitigation actions—the activities that are part of what we will call the “restoration economy.”

### Defining restoration

The Society for Ecological Restoration [8] broadly describes restoration as “the process of assisting the recovery of an ecosystem that has been degraded, damaged, or destroyed (Section 2).” Ecological restoration has traditionally been defined as an act of returning a system to an original state, and is distinguished from rehabilitation, which is more broadly defined as any act to improve the degraded state of the ecosystem [9]. Intact or “original” ecosystems will have both high structural and functional attributes compared to degraded systems. While ‘remediation,’ ‘reclamation,’ ‘enhancement,’ and ‘mitigation’ are also activities performed on degraded ecosystems, the final outcome of these activities is an alternative state or partial recovery of an original state.

For our purposes, we define restoration as any combination of activities intended to result in ecological uplift, improve ecosystem health, and result in a functioning ecosystem that provides a suite of ecosystem services (i.e. the beneficial functions of ecological systems [10]). These activities may include conservation activities—such as the purchase of conservation easements, land acquisition, or transfer of water rights—only when such investments are a part of a larger restoration effort. See S1 File for discussion. By defining the restoration economy around the industries that contribute to these efforts, we inductively define restoration as being comprised of the set of economic activities that contribute to restoration, from project planning, engineering and legal services, to intermediate suppliers of inputs, to on-the-ground earthmoving, forestry, and landscaping firms that contribute to the ecological restoration process.

Ecological restoration as a set of economic activities does not consistently fit within any single traditional economic sector (e.g. biomedical devices, automobile manufacturing), since pertinent activities range from scientific research and project planning, to earth moving and tree planting. Our goal in defining ecological restoration for the purposes of this project is not a theoretical statement of what we accept or reject as restoration activities. Instead, we are looking to the literature for a definition that simply serves to delineate the universe of activities that we choose to include in our analysis. Therefore, we largely ignore the theoretical tensions between environmental protection and restoration activities (those that minimize future degradation vs. mitigate previous degradation).

Because standard public data sources do not collect data on restoration related work and because there is no standard industrial classification (e.g. NAICS) for this sector, it is very difficult to study using traditional economic assessment techniques. Collaborative projects that involve federal, state and local partners from the public and private sectors, along with diverse funding sources and complex implementing agencies and programs further complicate tracking efforts and efforts to delineate industry activities. The variety of programs, funding sources, and implementing agencies makes for a complex national restoration industry that is difficult to delineate. It is important to note that American law treats the term ‘restoration’ differently in different contexts, and within different agencies. The terms restoration, rehabilitation, remediation, re-establishment, and reclamation are often used interchangeably in policy [11, 12], although they are often used in scientific discussions to define separate and distinct activities [9, 13].

### The green and restoration economies

Efforts now document an emerging, domestic “green economy,” which has seen rapid job growth in renewable energy production [14], energy efficient construction [15], and green goods and services industries [16]. However, ecosystem restoration often been excluded from “green” economy accounting, even though evidence now suggests the presence of a coherent restoration sector that increases the quality of public environmental goods [17–20], contributes to national economic growth and employment [21] and stimulates economic activities in a wide variety of other industries [22, 23]. Exclusion of ecological restoration is likely due to the complex economic inter-linkages and drivers of restoration demand. As a result, recent efforts to specifically assess the restoration industry have been relatively small-scale, focusing on a limited set of programs, specific projects, localized geography (local or state level), and individual funding sources [18, 19, 22, 24–26].

### Our current understanding of the restoration economy

Previous work [27–29] has documented the factors that create demand for restoration. These factors include:

1. Regulatory mechanisms (e.g. US Clean Water Act Section 404 or U.S. Endangered Species Act Section 7/10; [30, 31]) that mandate or incentivize public and private investment in restoration to offset development activities;
2. Public procurement of restoration through programs that contract directly with restoration providers. Examples include the U.S. Department of the Interior’s restoration efforts in the National Wildlife Refuge System [32];
3. Regional initiatives (e.g. the Chesapeake Bay Program [33 U.S.C. § 1267] or Louisiana’s Coastal Protection and Restoration Program [33]) that are enabled through a synthesis of legislation and partnerships at different levels of government;
4. Internal government agency policies—e.g. [34, 35]—that require or allow for regular agency activities (e.g. habitat management) to be carried out in a more sustainable or restorative manner, and;
5. Private investments by foundations, non-profits, corporations and institutions as a way to increase sustainability or meet corporate social responsibility goals (e.g. property owners in Philadelphia choosing to convert parking lots into natively-planted bio-swales planted in order to lower stormwater bills [36]).

What we currently know about the restoration industry at local scales is quite revealing; the employment effects of individual restoration projects may actually be greater than those in the oil and gas industry, which only supports about 5.2 jobs per $1 million invested [37, 38]. We can put this in context when we see evidence that school and gas pipeline construction result in ~19.2 and 21.8 jobs per $1 million invested, respectively [38]. Studies [21–23] show that restoration supports as many as 33 jobs per $1 million invested (this value spans a range between 6.8 and 39.7 based on location, geographic scale, and restoration type [29]) with an economic output multiplier of between 1.6–2.6 (multiplier for total economic output from investments), and an employment multiplier of between 1.5 and 3.8 (the number of jobs created for every restoration job). Both of these multipliers are well within the range of several other industries, including oil and gas [39], crop and livestock agriculture [38], and outdoor recreation [40]. An important caveat to mention here is that employment multipliers are not the only possible metric to compare policy alternatives. Specifically, a complete cost-benefit analysis would also examine the amount of value added per job created across alternatives.

Currently, public and private investments linked to compensatory mitigation have been conservatively been estimated at $3.8 billion per year (Environmental Law Institute 2007). More recent estimates [29] estimate average federal appropriations for restoration-related programs at $1.9 billion per year (2011–2013). Non-profit investments in natural resources and wildlife preservation and protection are estimated to exceed $4.3 billion annually [40], and private sector investments in the U.S. compensatory mitigation industry total an estimated $1.3 to $4.0 billion annually [27, 28]. Restoration projects also tend to create localized employment benefits [25, 26, 41], while creating relatively well-paying jobs compared to average wages; similar to the construction industry at large, however, significant inter-annual fluctuations and seasonality are major factors [26].

While these studies tell an important, localized story, the extent of these activities and benefits are not yet well understood at a national level. The goal of this paper is to provide a national-scale accounting of the size and scope of the restoration economy in terms of employment, value added and overall economic output on a national scale. We conducted a national survey of businesses that participate in restoration work in order to a) estimate the total sales and number of jobs directly associated with the Restoration Economy, and, b) provide a profile of this nascent sector in terms of type of restoration work, industrial classification, workforce needs, and growth potential. We used survey results as inputs into a national input-output model (IMPLAN 3.1) in order to estimate the indirect and induced economic impacts of restoration activities.

## Methods

To assess the overall economic impact restoration economy we developed an original survey of firms and organization engaged in restoration work. The survey was approved under the University of North Carolina at Chapel Hill's Institutional Review Board (UNC IRB #13–1872) and written consent was obtained from all respondents. The key goals of our empirical analysis were to a) provide a broadly representative national picture of the restoration economy, b) estimate the total sales and employment of restoration firms, and c) describe restoration work as segmented by standard industrial classification measures and type of restoration work (e.g. wetland mitigation, forest restoration).

### Defining the Universe(s) of restoration actors

Since we are studying and describing a portion of the U.S. economy that is emerging and has not been consistently cataloged, a key problem for our survey approach was to define the universe of potential economic actors engaged in restoration (see S2 File). We defined two sampling frames in which to execute our survey. The first—a *publicly-induced* restoration firm sample—captures the set of private and non-profits establishments that are funded either by direct federal procurement or through work mandated by public laws such as the Clean Water Act (33 US Code §1344; see [30]), or similar legislation that requires or induces mitigation. As a proxy for this full, unknown universe of actors we used the 2012 database of government contractor firms listed on USASpending.gov, limiting the sample to federal agencies known to be involved in restoration work (see S6 File for a full list of target agencies) and in industries related to restoration work (*n = 5*, *805* firms), thereby yielding a “potential” public sample.

The second strategy we used for universe identification aims to capture restoration actors who work for *private* sector initiated restoration projects. Through literature review and listserves from industry associations (e.g. the National Mitigation Banking Association), we collected information on an additional 550 total “potential” survey respondents.

### Survey development and goals

We administered a short, web-based survey in February and March 2014. Screening questions helped us determine an accurate sample response rate, given that our sampling design meant only a fraction of survey respondents would likely be engaged in restoration work (22 percent). This screening indicated a ‘public’ sample response rate of 11.5 percent (*n = 148*). Contrasting this, the sample of firms engaged in private sector derived mitigation indicated a higher likelihood of restoration work (72 percent), yielding an adjusted response rate of 25.6 percent (*n = 102*). We used each sample response rate to calculate separate sample-specific frequency weights; overall, the survey yielded 250 valid responses and an overall response rate of 14.8 percent. While lower than some previous restoration survey efforts (e.g. [42]), this response rate is in line with rates seen in other surveys that ask sensitive business questions [43]. While we have no known population to which we can compare our sample, we are confident that our survey is broadly representative. The survey respondents include firms that perform different roles (defined in [44]) in each major type of restoration work (based on restoration firm survey by US Geological Survey described in [45]) and the distribution of establishments across NAICS codes corresponds to findings from a previous literature review and survey of public restoration programs [29].

### Development of IMPLAN inputs for input-output modeling

In order to produce this level of economic output, restoration firms need to purchase input materials and services from other sectors of the economy (e.g. construction equipment, tools, computers, specialized services, etc.). Thus, other sectors are stimulated, or supported, indirectly from the direct sales of restoration firms (i.e. *indirect impact)*. Finally, workers employed directly by restoration related firms and indirectly in other sectors that sell inputs to restoration firms, spend their earnings on the typical variety of consumption goods and services needed to support their households (i.e. *induced impacts)*.

While the direct figures were derived from our survey, we used IMPLAN 3.1 (IMpacts for PLANing; Minnesota Implan Group, http://www.implan.com/) —an industry-standard input-output modeling software and data package—to calculate the indirect and induced impacts. While IMPLAN and similar input-output software packages are typically used to analyze the multiplier effects of a change in new final demand in a given sector throughout the economy, our analysis describes the level of economic activity (i.e. output, jobs) that is supported by restoration work in a given year. Thus, our interpretation of IMPLAN analysis results addresses the question, “how many jobs would be lost if the restoration work was not conducted?”

As an input-output modeling system, IMPLAN relies on data from the Bureau of Economic Analysis and other federal statistical agencies to describe the purchasing relationships between all industrial sectors in the US economy. In addition, IMPLAN provides an estimate of the number of jobs needed in each industrial sector to produce a given level of output (i.e. the output per worker ratio). This figure varies widely based, among other factors, on the capital intensity and productivity of each industry. Thus, we needed to refine our direct sales input by breaking out the level of sales/output by each specific industry.

Having asked respondents to list their primary industry—selecting the North American Industry Classification (NAICS) code that best matched their company’s activity—we calculated the weighted sales related to restoration for each industry listed (*n = 41* unique industries). Table 1 below lists the sales figures by NAICS for the top 15 industries/sectors reported, which represent the vast majority of sales (96.1 percent). This observed sales pattern is consistent with previous efforts to catalog restoration demand drivers [29]. Specifically, there is a roughly even split between the scientific/engineering/design aspects of restoration and the physical construction/earth moving/agricultural related aspects.

**Table 1:** Top 15 industries within the restoration economy by estimated sales, 2014.

NAICS Code	Weighted Sales, 2014 ($)	% of Total
5413-Architectural, Engineering, and Related Services	3,503,743,019	36.4
1151-Support Activities for Crop Production	2,243,711,385	23.3
2379-Other Heavy and Civil Engineering Construction	973,838,746	10.1
9241-Administration of Environmental Quality Programs	735,183,230	7.6
92-Public Administration	446,796,438	4.6
54-Professional, Scientific, and Technical Services	287,147,818	3.0
5416-Management, Scientific, and Technical Consulting Services	235,379,875	2.4
2373-Highway, Street, and Bridge Construction	213,919,343	2.2
4884-Support Activities for Road Transportation	148,640,476	1.5
1141-Fishing	126,722,762	1.3
5419-Other Professional, Scientific, and Technical Services	116,645,288	1.2
2389-Other Specialty Trade Contractors	102,139,494	1.1
531-Real Estate	50,447,677	0.5
23-Construction	48,741,430	0.5
1153-Support Activities for Forestry	21,560,834	0.2
Other Industries	229,483,090	3.9

### Limitations and clarifications

Before turning directly to survey results and findings, it is important to note several factors which we do not account for and thus limit the scope of our research design. First, our analysis is not intended to be a full cost-benefit analysis of restoration-related legislation in that we do not attempt to quantify the economic cost of forgone development activity or the price increase that may occur due to environmental regulations. Since significant policy research has already focused on the cost side (cf. [3, 7, 46]), we instead explicitly focus on a high-level valuation of the benefits of restoration work. Second, in focusing on readily quantifiable economic outcomes such as jobs, value-added, and output, we are selecting a relatively narrow scope of economic benefits that result from restoration. In particular, we do not account for the benefits of restored ecosystems that accrue from renewed ecosystems goods and services (e.g. flood prevention; [17, 47]).

## Results

### Descriptive statistics: Who is in the restoration economy?

Survey responses were nationally distributed, not only in terms of where firms were headquartered, but also in terms of where they conducted restoration activities. Each firm was asked to select the states where they have engaged in restoration projects. All 50 states were covered with the top five states being California, Virginia, Florida, Texas, and North Carolina; North Dakota (see S5 File) had the lowest number of respondents (*n = 10*) performing restoration work within the state.

Median firm size consisted of 13 full time employees and 3 part-time employees. The distribution of employment size was highly right-skewed (mean employment size was 807), indicating that there are some very large companies engaged in restoration work. However, this does not indicate that their entire workforce is engaged in restoration. We control for this possibility in our estimate of direct impacts by asking respondents about the share of their sales that is derived from restoration. Despite the rapid growth of restoration activity, just over half of restoration establishments have been in business over 20 years (*n = 103/199*). This indicates both the presence of a well-established set of firms, as well as the emergence of firms that are entering the restoration sector to meet new demand after having been established in other industries.

**Fig 1:**
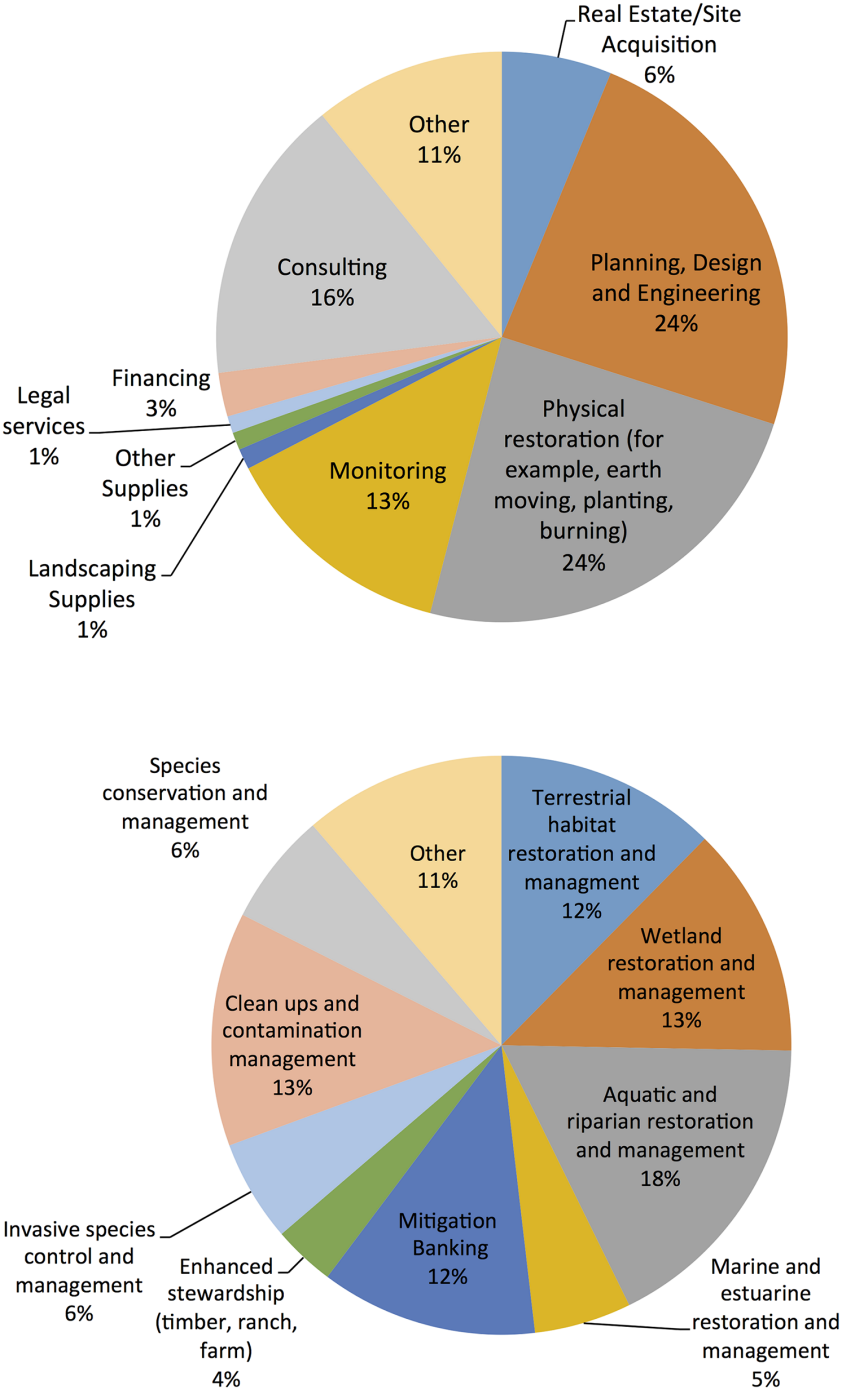
(A) Distribution of respondents by business function within the restoration economy. (B) Distribution of respondents by type of restoration work.

We can also describe the broader restoration sector by both the specific business function and type of restoration work of each establishment. The largest segments of restoration work involve 1) planning, design, and engineering activities and 2) physical restoration—the actual earth moving and site construction (Fig 1a). Survey respondents were evenly distributed in terms of the type of restoration work conducted, with wetland restoration (13%) and aquatic and riparian restoration (18%) representing the largest categories (Fig 1b). This likely indicates the role of the Clean Water Act’s Section 404 compensatory mitigation requirements in inducing restoration work [30].

In terms of economic output, the mean annual revenue from restoration work across our sample was $11.65 million, and the plurality of respondents indicated that restoration related revenue has increased over the past five years (57%), compared to declined (26%) or remained the same (16%). Below, we use reported sales from restoration related work by each respondent’s reported industrial sector as the basis for estimating the total impact of the restoration economy in the United States.

### IMPLAN modeling

Based on our weighted survey results, we estimate that within the last year, the restoration economy as a whole has produced $9.47 billion in economic output. This figure includes the value of all sales or revenue to firms engaged in all aspects of restoration work (i.e. *direct impact*), from the environmental scientists and engineering companies which plan a wetland restoration project, to the construction firms hired to complete the work, to the greenhouses and nurseries that grow plants. This activity directly generates 126,111 jobs each year and approximately $6.27 billion in labor income (i.e. wages and benefits). The average labor income per direct job was $49,734 in 2014 dollars, which represents a figure close to the median annual wage in the U.S. This direct restoration activity results in $6.29 billion in value added in the U.S. economy. It is important to focus on total output per full-time job as well as the total count of employment, and to put this figure in context because this gives a sense of the opportunity cost of the labor employed in restoration. Our analysis indicates that restoration activity generates approximately $75,170 in output per job. While this figure is lower than some highly capital intensive industries like oil extraction and manufacturing, it is only slightly smaller than construction ($111,722) and is greater than retail ($58,836), which are some of the sectors most impacted by land development regulations. Based on our survey size, we calculate a 6.2 percent margin of error for our survey responses at 95 percent confidence (see S3 File).

Beyond the direct impact, the restoration economy also supports additional employment and economic output through indirect spending (i.e. spending by direct businesses on inputs) and induced (i.e. household) spending. As indicated in Table 2 below, the indirect effect represents an additional 26,444 jobs and $4.61 billion in output, while 68,843 jobs and $10.76 billion in output are generated through household spending. All included, we estimate that the ecological restoration economy generates ~221,000 jobs and ~$24.86 billion in economic output.

**Table 2:** Overall annual economic impact of restoration economy.

Impact Type	Employment	Labor Income	Value Added	Gross Output
**Direct Effect**	126,111	$6,272,130,931	$6,293,032,304	$9,479,980,786
**Indirect Effect**	26,444	$1,615,165,988	$2,556,810,292	$4,615,797,176
**Induced Effect**	68,843	$3,520,387,488	$6,292,819,878	$10,762,860,487
**Total Effect**	221,398	$11,407,684,407	$15,142,662,473	$24,858,638,449

Rows sum vertically, but do not sum horizontally; ‘value added’ calculations include labor income and firm profit, while ‘economic output’ additionally includes the costs of inputs (i.e. cost of goods sold).

### Precision and context of estimates

It is important to note that our employment estimate of 126,111 jobs is derived from the sales inputs ($9.478 billion) observed in our survey. Specifically, we rely on IMPLAN data on output-per-worker ratios for the specific direct industries involved in restoration. Thus, while the IMPLAN results give a figure with a high degree of precision, this figure should be interpreted as an approximate number. For the sake of preventing redundancy in the output tables, we do not report the range of estimates since it would be a simple scaling up and down on the direct input figures. For example, if we apply the margin of error of +/- 6.2% to our direct employment estimate of 126,111 we would have a range from 118,279 to 133,943.

There are several factors that could generate bias in our employment estimates. First, the underlying data for the output-per-worker ratios that IMPLAN provides are derived from national data sources including the BEA and the Economic Census, each of which are subject to sampling error. In addition, the firms engaged in restoration may differ in their capital intensity or wage rate from the national average in a given IMPLAN sector (see S4 File for more information).

**Fig 2:**
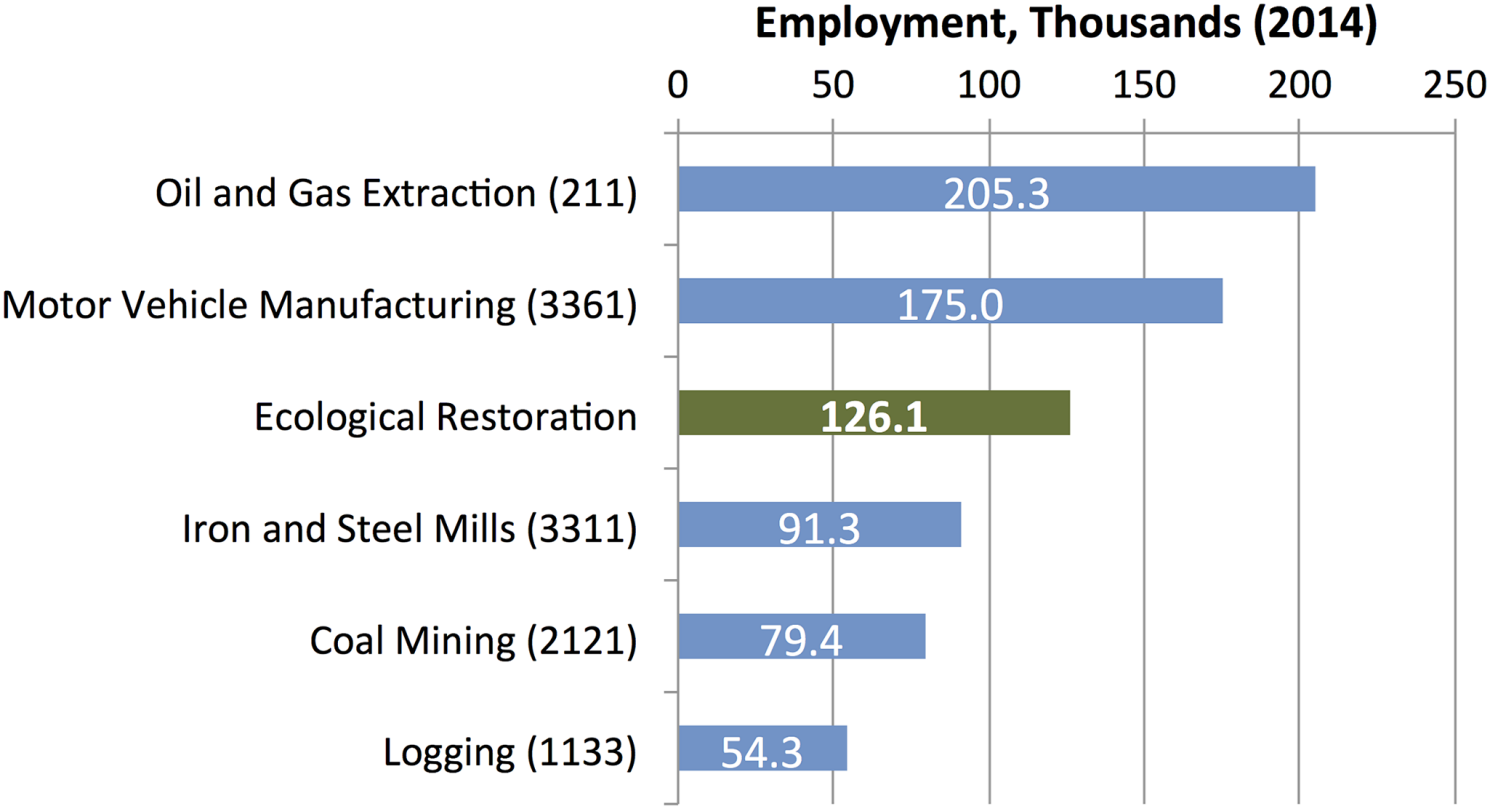
Direct jobs in ecological restoration and selected carbon intensive industries, 2014. Restoration employment figures from authors’ analysis of survey data. All other industry employment data are from U.S. Bureau of Labor Statistics (BLS), Current Employment Statistics Program (Jan 2014).

Given the approximate nature of our employment estimates, it is perhaps preferable to put our ‘ballpark’ estimate of restoration-related employment in the context of other well-known industries in the U.S. In Fig 2 below, we compare our direct jobs estimate to five other industries, which are often associated with carbon-intensive energy use or environmentally sensitive resource uses.

Our estimate of employment generated by ecological restoration compares favorably with several industries, which are often considered essential to our national competitiveness. For example, we find that there are more workers directly employed in restoration than coal mining, logging, or steel production. To give a comparison, the oil and gas extraction industry—not including related services—has less than twice the workers that are directly employed in the restoration economy.

### Industry and fiscal impacts

While the overall economic of restoration activity supports approximately 221,000 jobs including the direct, indirect and induced effects, these jobs are spread out across various industry sectors within the U.S. economy. Table 3 lists the top twenty industry sectors by total employment. Not surprisingly the top industries are those that are directly supported by restoration work itself, including support activities for agriculture and forestry, architectural, engineering and related services, and environmental and other technical consulting services. These three sectors represent nearly 90 percent of the direct employment and half of the total jobs supported by the restoration economy.

**Table 3:** Employment impacts by industry, top 20 ranked by total employment 2014.

IMPLAN Sector Description	Direct	Indirect	Induced	Total	Share of Total
**Support activities for agriculture and forestry**	69,640	196	224	70,059	32%
**Architectural, engineering, and related services**	27,503	1,992	202	29,697	13%
**Environmental and other technical consulting services**	12,236	361	110	12,707	6%
**Food services and drinking places**	0	1,792	6,725	8,517	4%
**Construction of other new nonresidential structures**	8,268	0	0	8,268	4%
**Employment services**	0	3,303	1,460	4,763	2%
**Real estate establishments**	311	850	2,949	4,110	2%
**Offices of physicians, dentists, and other health practitioners**	0	0	3,064	3,064	1%
**Private hospitals**	0	0	2,990	2,990	1%
**Wholesale trade businesses**	0	698	2,151	2,849	1%
**Other Federal Government enterprises**	2,602	12	49	2,663	1%
**Scenic and sightseeing transportation and support activities for transportation**	2,036	183	195	2,414	1%
**Nursing and residential care facilities**	0	0	2,110	2,110	1%
**Services to buildings and dwellings**	0	976	1,058	2,034	1%
**Securities, commodity contracts, investments, and related activities**	0	504	1,508	2,012	1%
**Retail Stores—General merchandise**	0	69	1,913	1,982	1%
**Retail Stores—Food and beverage**	0	71	1,870	1,940	1%
**Commercial Fishing**	1,785	1	5	1,791	1%
**Civic, social, professional, and similar organizations**	225	397	1,047	1,670	1%

Key sectors that are stimulated through indirect (i.e. business to business) spending are ‘employment services,’ which provides temporary labor, wholesale trade, and other engineering services. There are also several sectors which rank high in terms of job creation due primarily to spending by households, which received labor income from both the directly and indirectly supported sectors. These ‘residentiary’ sectors included food services and drinking places (so-named by the US Bureau of Labor Statistics), doctor’s offices, hospitals, and real estate establishments. These sectors reflect the typical household spending patterns of all workers.

Finally, our IMPLAN analysis yields a broad measure of the fiscal impacts of restoration work. Specifically, IMPLAN calculates an estimate of the total local, state and federal tax revenue generated by all economic activity generated through the direct restoration work. This estimate includes all sources of revenue from federal income taxes and social insurance payments, to state corporate taxes, to local fees and property taxes. Ultimately, the overall economic impact of $24.8 billion supports approximately $1.02 billion for local and state coffers and an additional $2.13 billion for the Federal government. It is important to note that these tax impacts are only measurements of revenue collected because of the restoration work and is not net of any public procurements that pay for restoration (i.e. a full fiscal cost-benefit study). However, as we note in our discussion of demand drivers, only a small amount of restoration work is directly funded by government, compared to private sector activity that is induced by regulation or other motives.

## Discussion

The design-construction linkage dominating the employment within the restoration economy is a relatively unique feature of this industry, which also has important impacts on the workforce requirements of this emerging sector. In particular, the longevity of restoration firms responding to our survey indicates a strong presence of mature companies that are looking to restoration work to expand their business. Interpreted from an economic development point of view, this results in a multi-dimensional labor demand function for workers with both limited post-secondary education (e.g. in construction and landscaping industries), with a bachelor’s degree and also those with an advanced degree in engineering.

It appears that the sector is growing; this supports previous and repeated assessments of biodiversity markets [27, 28, 48] and watershed investments and payments [49–51] that suggest a global trend of increasing investments in ecological restoration due to growing markets for ecosystem services. Our work suggests, however, a domestic sensitivity to regulations that require ecological restoration, as well as potential sensitivity to commodity prices (e.g. California carbon market; [52]) that could affect future sector expansion.

In this article, we assess the size of the restoration economy as a means of determining the level of employment and economic output that is produced as we endeavor to restore damaged ecosystems. Much as estimates of the costs of environmental policies focus on the job reductions in industries experiencing regulatory requirements, our analysis focuses singularly on the jobs produced by ecological restoration, and does not attempt to produce a full cost-benefit analysis. That is, we do not consider the opportunity cost of investments in other industries incurred due to investments in restoration activities. The work of quantifying the size and scope of the restoration economy parallels a burgeoning field of research on the so-called “green economy” generally.

A growing number of studies have identified “green” growth and job creation in renewable energy production [14], energy efficient construction [15], and green goods and services industries [16]. Marking the official recognition of this important sector, the U.S. Bureau of Labor Statistics’ instituted a unique *Green Goods and Services* survey, which found that the “green” economy accounted for 3.4 million U.S. jobs in 2011, with the vast majority of jobs in the private sector [53]. The green economy has also been recognized as a potential source of innovation that drives the broader economy [54]. This study represents a first step towards quantifying the restoration industry as a piece of the broader green economy.

## Supporting Information

S1 File Notes on the definition of ‘ecological restoration’. (PDF)

S2 File Sampling technique. (PDF)

S3 File Survey instrument, delivery, and response rate. (PDF)

S4 File Development of IMPLAN Inputs. (PDF)

S5 File Survey questions. (PDF)

S6 File List of federal agencies targeted. (PDF)
