# Supplementary material for: Estimating the Size and Impact of the Ecological Restoration Economy
Source: PLoS One. 2015 Jun 17;10(6):e0128339. doi: 10.1371/journal.pone.0128339 (PMC4470920; doi:10.1371/journal.pone.0128339)
Supplement: S1 File — (PDF) [file pone.0128339.s001.pdf]

Supporting Information S1: Notes on the definition of 'ecological restoration'

Rather than parse a specific definition of ecological restoration, many previous economic impact studies define the scope of the restoration industry by the activities and programs that are included in and excluded from their analysis. The choice to exclude a particular activity, such as sustainable forest management, may hinge more on data availability than whether or not the activity constitutes restoration (e.g. [1]). Similarly, the choice to include a particular activity, such as site remediation, might occur when the activity cannot easily be separated from a larger restoration project [2,3].

Similarly, the choice to include or exclude *conservation* activities in the *restoration* category is one for which there is no consensus. Restoration activities exist within the broader context of conservation when conserving an ecosystem or species hinges on restoring the remains of degraded habitat or populations [4]. Likewise, conservation activities become a necessary part of restoration when the trajectory of improved ecosystem health depends on its long-term protection.

1. Baker M (2004) Socioeconomic Characteristics of the Natural Resources Restoration System in Humboldt County, California. Taylorsville, CA: Forest Community Research.
2. Shropshire R, Wagner B (2009) An Estimation of the Economic Impacts of Restoration in Montana. Helena, MT: Research and Analysis Bureau, Montana Department of Labor and Industry.
3. DOI (2012) The Department of the Interior's Economic Contributions, FY 2011. Washington, D.C.: U.S. Department of the Interior, Office of Policy Analysis.
4. Hobbs RJ, Harris JA (2001) Restoration Ecology: Repairing the Earth's Ecosystems in the New Millennium. Restoration Ecology 9: 239-246.
