# Supplementary material for: Estimating the Size and Impact of the Ecological Restoration Economy
Source: PLoS One. 2015 Jun 17;10(6):e0128339. doi: 10.1371/journal.pone.0128339 (PMC4470920; doi:10.1371/journal.pone.0128339)
Supplement: S2 File — (PDF) [file pone.0128339.s002.pdf]

Supporting Information S2: Sampling technique

The overall goal of our survey analysis was to generate a broad, nationally representative snapshot of the Restoration Economy and measure the total amount of economic activity in this sector and characterize its industrial make-up. Since we are studying and describing a portion of the U.S. economy that is emerging and has not been consistently cataloged, a key problem for our survey approach is to define the universe of potential economic actors engaged in restoration. We define the unit of analysis of potential actors to be made up on either private for-profit establishments, private non-profits, or public agencies directly engaged in restoration work. Since there is no consistent business listing or public industrial classification code for restoration work, we needed to develop a strategy of capturing as broad a universe of potential restoration actors as possible.

Based on literature review and analysis of demand drivers for restoration [1-3], we developed *publicly*- and *privately-induced* restoration firm samples as our strategies for creating an appropriate sample universe. As a proxy for the former, we used the 2012 database of government contractor firms listed on USASpending.gov. This publically available database is maintained by the federal government and is intended to show how federal tax dollars are spent and includes detailed information including contract size, location and industry characteristics for all companies that were hired as contractors or subcontractors to federal agencies.

We then used a series of limiting assumptions to narrow the full list of federal contractors to a “potential universe” of restoration actors. First, we limited our analysis to contractors of federal agencies that were most likely to be involved with restoration

work, including the Forest Service, Army Corps of Engineers, and the Environmental Protection Agency (see Supp. Information 6 for a full list of target agencies). This resulted in a list of over 32,000 contractors or subcontractors. However, since many of these agencies hire contractors to provide goods or services that are completely unrelated to restoration (e.g. telecommunications, janitorial services, etc.), we further limited the list to companies with a principal NAICS code that was plausibly related to restoration work. We selected 351 out of 960 unique six-digit NAICS industry descriptions, which further limited our potential universe to approximately 12,000. Since the federal database also included a unique identifier (DUNS), we then matched this list to a private sector business-listing database operated by Dun and Bradstreet Inc. (Hoovers.com), which allowed us to identify contact information for each firm. Ultimately, we ended up with a list of 5,805 companies with email addresses that comprise our "potential" public sample.

For our *privately-induced* restoration firm survey sample, we used previous literature reviews and discussions of demand drivers to determine that a large portion of this part of the restoration sector is driven by mitigation banks. Thus we used contact lists maintained by the National Mitigation Banking Association, which included contractors hired by hundreds of mitigation banks that are current and former members of NMBA.

1. BenDor TK, Livengood A, Lester TW, Davis A, Yonavjak L (In Press) Defining and Evaluating the Ecological Restoration Economy. Restoration Ecology In Press.
2. Madsen B, Carroll N, Moore Brands K (2010) State of Biodiversity Markets Report: Offset and Compensation Programs Worldwide. Washington, D.C.: Ecosystem Marketplace.
3. ELI (2007) Mitigation of Impacts to Fish and Wildlife Habitat: Estimating Costs and Identifying Opportunities. Washington, D.C.: Environmental Law Institute.
