# Supplementary material for: Estimating the Size and Impact of the Ecological Restoration Economy
Source: PLoS One. 2015 Jun 17;10(6):e0128339. doi: 10.1371/journal.pone.0128339 (PMC4470920; doi:10.1371/journal.pone.0128339)
Supplement: S3 File — (PDF) [file pone.0128339.s003.pdf]

Supporting Information S3: ***Survey instrument, delivery, and response rate***

Our final survey instrument, which was pre-tested on *n=12* organizations cutting across a range of firm types, consisted of 20 questions and aimed to take approximately 10-15 minutes. The survey length was kept short to increase response likelihood [1]. The survey was delivered via e-mail (using the Qualtrics survey delivery software) and was open for response between February 1st and March 1st, 2014. Three reminders were sent to respondents and participation was induced through an optional raffle entry to win one of 10 small Amazon gift cards and gift baskets.

While the instruments asked respondents a variety of questions regarding their company/organizations characteristics—including type of restoration work engaged in, key driving legislation, geographic focus, and workforce characteristics—key questions for our purposes concerned the company's annual sales. Since some firms—especially those engaged in construction or earth moving activities—likely work on projects that are unrelated to restoration, we also asked each respondent to estimate the share of their annual sales that were derived from restoration work (Supp. Information 5 contains our survey questions). Through these two values, we were able to determine relative firm sales associated with restoration activities.

However, determining the accurate sample response rate was a critical step in our analysis since it will be used to develop frequency weights for final calculations of the overall size of the restoration economy. As indicated above, we do not know the exact size of the sample population. Thus, the first challenge was to limit the "potential" universe pools described above to only those firms that are actively engaged in restoration work. Thus, the first question we asked each respondent was "Has your company ever participated in any aspect of environmental restoration work, or worked with an environmental restoration company?" If

respondents answered “no”, then the exited the survey. We used this question to limit the size of the universe in each sample (public, private).

Specifically, in the public sample we received 671 total responses, of which only 148 (22 percent) indicated that they actually conducted work in the restoration economy. We then applied this share to the full “potential” universe size of 5,805 to come up with a adjusted universe size of 1,280. Thus our response rate for the full survey in the public sample was 148/1280 or 11.5%. The private sample, not surprisingly, was better targeted to establishments that were actually engaged in restoration work. However, even here only 72 percent of respondents indicated participation in restoration work. Thus, the adjusted universe size for the private sample was 398, of which 102 respondents completed the survey for a response rate of 25.6 percent. The steps we use to limit the universe of potential restoration firms represent a conservative approach, since they lead to much lower frequency weights, thereby leading to low levels of inflation of total sales estimates. In total, the survey yielded 250 valid responses and an overall response rate of 14.8 percent.

With a sample size of 250 total respondents, we can provide a broad assessment of the accuracy of our direct inputs by calculating the simple margin of error for the survey as a whole. Assuming that we drew a simple random sample for the universe of restoration actors, then we can calculate the margin of error (assuming a 95 percent confidence level) as $\frac{1.96}{2*\sqrt{250}}$, which is 0.062 or 6.2%.

1. Dillman DA, Smyth JD, Christian LM (2008) Internet, Mail, and Mixed-Mode Surveys: The Tailored Design Method (3rd Edition). Hoboken, NJ: Wiley.
