# Supplementary material for: Estimating the Size and Impact of the Ecological Restoration Economy
Source: PLoS One. 2015 Jun 17;10(6):e0128339. doi: 10.1371/journal.pone.0128339 (PMC4470920; doi:10.1371/journal.pone.0128339)
Supplement: S4 File — (PDF) [file pone.0128339.s004.pdf]

**Supporting Information S4: Development of *IMPLAN Inputs***

We use the results of our national survey to estimate the total amount of economic activity generated *directly* by firms and organizations engaged in restoration. We combine three key questions to develop the direct inputs; 1) the total annual sales reported by each respondent; 2) the reported share of sales from restoration-related project; and 3) the respondent's primary industry. Respondents were asked to list their sales figures either as a directly inputted figure or as a range. We assumed a linear distribution within each range and used the midpoint of the reported range as the imputed value for each record. For the top range, we used the lowest threshold value. Respondents reported the percentage from restoration directly. Each respondent was asked to select their North American Industry Classification System (NAICS) code on a hierarchical basis, starting at the 2-digit super-sector level, followed by the 3-digit sector level and finally a 4 digit industry level. Since some respondents were not able to specify their detailed NAICS code we based our calculations on the lowest reported level.

The final step in developing the inputs for IMPLAN 3.1 – IMpacts for PLANing, an industry-standard economic impact modeling software package – was to assign IMPLAN specific codes to each NAICS code. We used the NAICS-IMPLAN bridge provided by IMPLAN (Minnesota Implan Group (MIG), Inc; http://www.implan.com) to re-assign sales based on IMPLAN's own proprietary sectoring scheme. All sales values were inputted in 2014 dollars.

## Table A4- Direct Sales by NAICS code and Assigned IMPLAN Sector

| NAICS4 Reported From Survey | Weighted Sales ($) | Assigned IMPLAN Sector |
|---|---|---|
| 5413-Architectural, Engineering, and Related Services | 3,364,796,513 | 369-Architectural, engineering, and related services |
| 1151-Support Activities for Crop Production | 2,154,733,439 | 19-Support activities for agriculture and forestry |
| unknown | 1,145,619,588 | *reallocated to observed dist.* |
| 2379-Other Heavy and Civil Engineering Construction | 935,219,620 | 36-Construction of other new nonresidential structures |
| 9241-Administration of Environmental Quality Programs | 706,028,369 | 375-Environmental and other technical consulting services |
| 5416-Management, Scientific, and Consulting services | 226,045,511 | 375-Environmental and other technical consulting services |
| 2373-Highway, Street, and Bridge Construction | 205,436,031 | 36-Construction of other new nonresidential structures |
| Don't Know (please specify) | 163,887,116 | *reallocated to observed dist.* |
| 4884-Support Activities for Road Transportation | 142,745,902 | 338-Scenic and support activities for transportation |
| 1141-Fishing | 121,697,370 | 17-Fishing |
| 5419-Other Professional, Scientific, and Technical Services | 112,019,534 | 380-All other miscellaneous professional and technical services |
| 2389-Other Specialty Trade Contractors | 98,088,989 | 36-Construction of other new nonresidential structures |
| 1153-Support Activities for Forestry | 20,705,805 | 19-Support activities for agriculture and forestry |
| 5417-Scientific Research and Development Services | 12,142,831 | 376-Scientific research and development services |
| 5242-Agencies, Brokerages, and Other Related Activities | 11,702,128 | 358-Insurance agencies, brokerages, and related activities |
| 2383-Building Finishing Contractors | 11,679,210 | 36-Construction of other new nonresidential structures |
| 5182-Data Processing, Hosting, and Related Services | 9,083,830 | 352-Data processing, hosting, and related services |
| 6113-Colleges, Universities, and Professional Schools | 8,878,339 | 392-Junior colleges, colleges, universities, and prof. schools |
| 5629-Remediation and Other Waste Management Services | 6,488,450 | 390-Waste management and remediation services |
| 3344-Semiconductor and Other Electronic Component Mfg. | 5,190,760 | 247-Other electronic component manufacturing |
| 1119-Other Crop Farming | 3,957,955 | 10-All other crop farming |
| 5411-Legal Services | 2,925,532 | 367-Legal services |
| 1114-Greenhouse, Nursery, and Floriculture Production | 2,755,428 | 6-Greenhouse, nursery, and floriculture production |
| 2362-Nonresidential Building Construction | 2,595,380 | 36-Construction of other new nonresidential structures |
| 8113-Commercial and Industrial Machinery | 2,530,496 | 417-Commercial and industrial machinery |
| 4442-Lawn and Garden Equipment and Supplies Stores | 1,310,667 | 323-Retail - Building material and garden supply |
| 5616-Investigation and Security Services | 1,297,690 | 387-Investigation and security services |
| 5313-Activities Related to Real Estate | 877,660 | 360-Real estate |
| 1131-Timber Tract Operations | 389,307 | 15-Forest nurseries, forest products, and timber tracts |
| 2361-Residential Building Construction | 43,256 | 38-Construction of other new residential structures |
| 5322-Consumer Goods Rental | 12,977 | 363-General and consumer goods rental |
