# Supplementary material for: Estimating the Size and Impact of the Ecological Restoration Economy
Source: PLoS One. 2015 Jun 17;10(6):e0128339. doi: 10.1371/journal.pone.0128339 (PMC4470920; doi:10.1371/journal.pone.0128339)
Supplement: S5 File — (PDF) [file pone.0128339.s005.pdf]

Supporting Information S5: ***Survey questions***

Restoration Economy Survey

Q1 Welcome, You have been selected to participate in a study about the restoration industry in the United States. The goal of this study is to estimate the national economic impacts of environmental restoration. Your responses and feedback are very important. All of your responses are completely confidential and voluntary. Your name will not be associated with any of your answers and all information will be kept strictly confidential. Your company name will only be used to ensure that the same company is not represented more than once in the survey results. This survey contains 18 questions, and will take about 15 to 20 minutes of your time. You may choose to exit the survey at any time. As a thank you for your time and help, you will be presented with the option to enter into a drawing for a gift basket containing a $50 Amazon gift card and other environmental gear. Directions for entering this drawing will be presented at the end of this survey. If you have any questions or concerns about this survey, please contact Dr. Todd BenDor, Associate Professor at the University of North Carolina, Chapel Hill, at bendor@ unc.edu.

Q2 Has your company ever participated in any aspect of environmental restoration work?
❍ Yes (1)
❍ No (2)
If No Is Selected, Then Skip To End of Survey

Q4 Please provide some basic information about your company or organization below.   What is the name of your company or organization?

Q5 What state are you located in? (If you have multiple locations, please select the location of the company headquarters.) [List of States provided, along with “Outside the United States]

Q6 What year was your company or organization founded?

Q26 What is your primary industry (Please indicate the North American Industry Classification (NAICS) code that best describes your line of work, if known)

Sector (1)
Industry (2)
Detailed industry (3)

Q7 Please tell us about your company or organization's restoration work, below.   What type of services does your company or organization provide on environmental restoration projects? (If you provide more than one type of service, please indicate the approximate share of each by dragging the bar to the right).
______ Real Estate/Site Acquisition (1)
______ Planning, Design and Engineering (2)
______ Physical restoration (for example, earth moving, planting, burning) (3)
______ Monitoring (4)
______ Landscaping Supplies (5)
______ Other Supplies (6)
______ Legal services (7)
______ Financing (8)
______ Consulting (9)
______ Other (10)

Answer If: What type of services does your company or organization provide on environmental restoration projects? (If you provide more than one type of service, please indicate the approximate share of each... Other Is Greater Than or Equal to: 25

Q25 Please describe the services you provide that fall into the "other" category:

Q8 What type of environmental restoration does your company or organization participate in? (If you participate in more than one type, please indicate the approximate share of each by dragging the bar to the right). [Respondents asked to give percentages of work devoted to these areas]
______ Terrestrial habitat restoration and management (1)
______ Wetland restoration and management (2)
______ Aquatic and riparian restoration and management (3)
______ Marine and estuarine restoration and management (4)
______ Mitigation Banking (5)
______ Enhanced stewardship (timber, ranch, farm) (6)
______ Invasive species control and management (7)
______ Clean ups and contamination management (8)
______ Species conservation and management (9)
______ Other (10)

Answer If: What type of environmental restoration does your company or organization participate in? (If you participate in more than one type, please indicate the approximate share of each by dragging the bar... Other Is Greater Than or Equal to 25

Q26 Please describe the type of environmental restoration your company or organization participates in that falls into the "other" category:

Q10 A critical aspect of this survey is to estimate the total economic impact of restoration work nationwide. Therefore it is essential that we ask about the total sales and employment levels at your company. All of your responses are completely confidential. What was the total revenue (sales) of your company or organization in the most recent fiscal year? Include all revenue whether it is restoration-related or not.

- ❍ under $100,000 (1)
- ❍ $100,000 - 200,000 (2)
- ❍ $200,000 - 500,000 (3)
- ❍ $500,000 - $1,000,000 (4)
- ❍ $1,000,000 - $5,000,000 (5)
- ❍ $5,000,000 - $10,000,000 (6)
- ❍ $10,000,000 - $20,000,000 (7)
- ❍ $20,000,000 - $40,000,000 (8)
- ❍ $40,000,000 - $60,000,000 (9)
- ❍ $60,000,000 - $80,000,000 (10)
- ❍ $100,000,000 - $250,000,000 (11)
- ❍ $250,000,000 - $500,000,000 (12)
- ❍ more than $500,000,000 (13)

Q11 What percentage of your total revenue (sales) in your company or organization is derived from environmental restoration work?
______ Percent of revenue from environmental restoration work (1)

Q12 How many full-time employees do you have?

Q13 How many part-time employees do you have?

Q14 Does your employment level change significantly throughout the year? (For example, if your company or organization hires seasonal employees to work on specific projects there would be a change in employment level over the course of one year.)

- ❍ Yes. It changes very significantly. (1)
- ❍ Yes. It changes somewhat significantly. (2)
- ❍ No. It does not change significantly. (3)

Answer If: Does your employment level change significantly throughout the year? (For example, if your company or organization hires seasonal employees to work on specific projects there would be a change in... Yes. It changes very significantly. Is Selected Or Does your employment level change significantly throughout the year? (For example, if your company or organization hires seasonal employees to work on specific projects there would be a change in... Yes. It changes somewhat significantly. Is Selected

Q15 What are the minimum and maximum number of people employed by your company or organization last year?

______ Minimum (1)
______ Maximum (2)

Q16 Please indicate the share of your total workforce within each level of educational attainment.

______ Less than high school graduate (1)
______ High school graduate or equivalent (GED) (2)
______ Some college or associate's degree (3)
______ Bachelor's degree (4)
______ Graduate or professional degree (5)

Q17 What is the primary driver of your company or organization's restoration-related work?

______ Clean Water Act Section 404 (1)
______ Endangered Species Act (2)
______ NRDA (3)
______ CERCLA (4)
______ Abandoned Mine Lands Program (5)
______ GOMESA (6)
______ USDA NRCS program (for example, EQIP, WHIP, wetland reserve program) (7)
______ Farm Service Agency program (for example, conservation reserve program) (8)
______ U.S. Fish and Wildlife Service program (for example, the landowner incentive program) (9)
______ NOAA program (for example, community-based restoration grants) (10)
______ Forest Service program (for example, stewardship contracting) (11)
______ Carbon/GHG offset markets (12)
______ Conservation banking credits (13)
______ Wetland/stream banking credits (14)
______ Other (15)

Q18 What type of organization typically hires your company or organization to do restoration work?

- ❍ Private company (1)
- ❍ Government agency (2)
- ❍ Non-profit organization (3)
- ❍ Multiple types (4)
- ❍ Other (5) ____________________

Q19 Where does your company engage in environmental restoration work?   (check all that apply) [List of states provided, along with “I do not work in the United States”]

Q20 Please tell us about the trends your company or organization is experiencing, below.   Over the last five years, has your company or organization's total revenue (sales):

- Increased (1)
- Decreased (2)
- Remained the same (3)

Q21 Over the last five years, has the share of your company or organization's total revenue (sales) that is related to environmental restoration:

- Increased (1)
- Decreased (2)
- Remained the same (3)

Q22 What are the largest barriers to your company obtaining more restoration related work?

Q23 Thank you! Thank you for taking the time to complete our survey. Your responses will help us to estimate the overall size of the restoration industry and the national economic impacts in terms of revenue and employment. Your company or organization's information will remain confidential.   If you wish to be entered into a drawing for a $50 Amazon gift card and other environmental gear, please enter your email address in the space below. You will be notified by email if you have been randomly selected for a prize.
