# Supplementary material for: Estimating the Size and Impact of the Ecological Restoration Economy
Source: PLoS One. 2015 Jun 17;10(6):e0128339. doi: 10.1371/journal.pone.0128339 (PMC4470920; doi:10.1371/journal.pone.0128339)
Supplement: S6 File — (PDF) [file pone.0128339.s006.pdf]

Supporting Information S6: ***List of federal agencies targeted***

As described in the methods section and in Supporting Information S2, we used publicly available contractor data list of establishments who received funding from the American Recovery and Reinvestment Act (ARRA) available from USASpending.gov. We narrowed our selection of potential contacts by selecting a subset of federal agencies and departments that were found to administer restoration related work, based on our previous research. Specifically, we selected on the following values from each of the following variables if it had *any* of the values (i.e. using an 'or' logic):

Variable: 'maj_agency_cat'
1100: EXECUTIVE OFFICE OF THE PRESIDENT
1200: AGRICULTURE, DEPARTMENT OF
1400: INTERIOR, DEPARTMENT OF THE
6500: FEDERAL MARITIME COMMISSION
6800: ENVIRONMENTAL PROTECTION AGENCY
6900: TRANSPORTATION, DEPARTMENT OF
7000: HOMELAND SECURITY, DEPARTMENT OF
9700: DEPT OF DEFENSE

Variable: 'mod_agency'

1100: EXECUTIVE OFFICE OF THE PRESIDENT
1205: USDA, OFFICE OF THE CHIEF FINANCI
12C2: FOREST SERVICE
12C3: NATURAL RESOURCES CONSERVATION SE
12D2: FARM SERVICE AGENCY
1330: NATIONAL OCEANIC AND ATMOSPHERIC
1422: BUREAU OF LAND MANAGEMENT
1425: BUREAU OF RECLAMATION
1434: GEOLOGICAL SURVEY
1435: BUREAU OF OCEAN ENERGY MANAGEMENT
1438: OFFICE OF SURFACE MINING, RECLAMA
1443: NATIONAL PARK SERVICE
1448: U.S. FISH AND WILDLIFE SERVICE
1450: BUREAU OF INDIAN AFFAIRS
1700: DEPT OF THE NAVY
2100: DEPT OF THE ARMY
5700: DEPT OF THE AIR FORCE
6500: FEDERAL MARITIME COMMISSION

6800: ENVIRONMENTAL PROTECTION AGENCY
6925: FEDERAL HIGHWAY ADMINISTRATION
6930: FEDERAL RAILROAD ADMINISTRATION
7008: U.S. COAST GUARD
7022: FEDERAL EMERGENCY MANAGEMENT AGEN

Variable: 'maj_fund_agency_cat'

1100: EXECUTIVE OFFICE OF THE PRESIDENT
1145: PEACE CORPS
1200: AGRICULTURE, DEPARTMENT OF
1400: INTERIOR, DEPARTMENT OF THE
6400: TENNESSEE VALLEY AUTHORITY
6500: FEDERAL MARITIME COMMISSION
6800: ENVIRONMENTAL PROTECTION AGENCY
6900: TRANSPORTATION, DEPARTMENT OF
7000: HOMELAND SECURITY, DEPARTMENT OF
9700: DEPT OF DEFENSE

Variable: 'contractingofficeagencyid'
1100: EXECUTIVE OFFICE OF THE PRESIDENT
12C2: FOREST SERVICE
12C3: NATURAL RESOURCES CONSERVATION SE
12D2: FARM SERVICE AGENCY
1330: NATIONAL OCEANIC AND ATMOSPHERIC
1422: BUREAU OF LAND MANAGEMENT
1425: BUREAU OF RECLAMATION
1434: GEOLOGICAL SURVEY
1435: BUREAU OF OCEAN ENERGY MANAGEMENT
1436: BUREAU OF SAFETY AND ENVIRONMENTA
1438: OFFICE OF SURFACE MINING, RECLAMA
1443: NATIONAL PARK SERVICE
1448: U.S. FISH AND WILDLIFE SERVICE
1450: BUREAU OF INDIAN AFFAIRS
1700: DEPT OF THE NAVY
2100: DEPT OF THE ARMY
5700: DEPT OF THE AIR FORCE
6500: FEDERAL MARITIME COMMISSION
6800: ENVIRONMENTAL PROTECTION AGENCY
6925: FEDERAL HIGHWAY ADMINISTRATION
6930: FEDERAL RAILROAD ADMINISTRATION
7008: U.S. COAST GUARD
7022: FEDERAL EMERGENCY MANAGEMENT AGEN
